# Temporal Attention as a Scaffold for Language Development

**DOI:** 10.3389/fpsyg.2016.00044

**Published:** 2016-02-02

**Authors:** Ruth de Diego-Balaguer, Anna Martinez-Alvarez, Ferran Pons

**Affiliations:** ^1^Institució Catalana de Recerca i Estudis AvançatsBarcelona, Spain; ^2^Cognition and Brain Plasticity Unit, Institut d’Investigació Biomèdica de BellvitgeBarcelona, Spain; ^3^Department of Basic Psychology, University of BarcelonaBarcelona, Spain; ^4^Department of Basic Psychology, Institute for Brain, Cognition and Behavior (IR3C), University of BarcelonaBarcelona, Spain

**Keywords:** language development, infancy, attention, temporal orienting, statistical learning, rule learning, morphosyntactic development, word segmentation

## Abstract

Language is one of the most fascinating abilities that humans possess. Infants demonstrate an amazing repertoire of linguistic abilities from very early on and reach an adult-like form incredibly fast. However, language is not acquired all at once but in an incremental fashion. In this article we propose that the attentional system may be one of the sources for this developmental trajectory in language acquisition. At birth, infants are endowed with an attentional system fully driven by salient stimuli in their environment, such as prosodic information (e.g., rhythm or pitch). Early stages of language acquisition could benefit from this readily available, stimulus-driven attention to simplify the complex speech input and allow word segmentation. At later stages of development, infants are progressively able to selectively attend to specific elements while disregarding others. This attentional ability could allow them to learn distant non-adjacent rules needed for morphosyntactic acquisition. Because non-adjacent dependencies occur at distant moments in time, learning these dependencies may require correctly orienting attention in the temporal domain. Here, we gather evidence uncovering the intimate relationship between the development of attention and language. We aim to provide a novel approach to human development, bridging together temporal attention and language acquisition.

## Introduction

Speech is a complex auditory stimulation. A single word in speech can be perceived as a sequence of phonemes, as a whole word, as a stem and a suffix or as having a specific meaning, depending on the level of processing. In order to face this complexity infants do not learn all of this information at once but rather in an incremental fashion. In particular, two main linguistic milestones -word segmentation and non-adjacent rule acquisition- appear in a sequential fashion. During the first months, infants are able to segment speech into words and recognize them; however, it is not until after the first year that they are able to understand and detect the subtle changes carried by different rule transformations (Gómez and Maye, 2005; Christophe et al., 2008). As a matter of fact, brain development in general is not uniformly distributed through infancy. Different brain structures and their white matter connections do not develop homogenously, nor do all cognitive functions develop at the same speed (Gogtay et al., 2004; Diamond, 2007). In particular attention shows also a developmental progression with different mechanisms arising at different moments of development. *Exogenous attention*, captured by salient events in the environment, is functional much earlier than *endogenous* *attention*, which allows for selecting which information to process and which to ignore (Posner and Cohen, 1984). The progressive general cognitive development may be seen as affecting all functions independently. However, cognitive functions do not work in isolation. In particular, the attentional system acts as a filter to any incoming stimulation, influencing perception, and therefore may affect learning, which suggests that the development of the attention system is likely to shape the way language is processed and how it develops along with the available attention resources.

Extensive literature has previously reported a link between attention and different aspects of language processing. Adult speakers of different languages tend to adapt the syntactic structure of their productions as a function of their focus of attention on the visual information they describe (Myachykov et al., 2005; Ibbotson et al., 2013; Tomblin and Myachykov, 2015). Similarly, focus and topicalization are naturally used to draw attention to relevant elements in the sentence (Jackendoff, 2002). Indeed, using focus to comprehend sentences activates left inferior parietal lobe (IPL) overlapping with the attention-orienting network used in visual attention (Kristensen et al., 2012). Even babies produce isolated words in an attempt to attract the listener’s attention to their own focus of interest (Jackendoff, 2002). This behavior is closely linked to the development of joint attention present at the end of the first year of life (Bruner, 1983; Carpenter et al., 1998; Brooks and Meltzoff, 2002). From joint attention interaction infants begin to link words with objects and events (Baldwin, 1991, 1993).

This previous literature converges with our proposal with the close link between attention and language, the source of the link is closely related in these studies to the communicative roots of language, given that attention is used as a tool to drive the listener’s attention to the focus of attention of the speaker (Smith et al., 2011). However, our proposal, in contrast, is intimately related to processing. The development of attention affects how the input is processed because it filters the input received, independently of the presence of an interlocutor and in the absence of a message to be transmitted. This is what shapes language learning from this point of view, in the same way as it shapes learning of other sources of information.

The present proposal presents an integrative approach of language acquisition, in which the powerful and dynamic interplay of exogenous and endogenous attention mechanisms allows infants to focus on different aspects of speech at different moments in development. In particular, during the initial stages of language acquisition, attention is captured by salient elements of speech, such as prosodic cues (e.g., pitch, rhythm, or pauses) because the infant perceptual system is guided by stimulus-driven attention. As months pass and endogenous attention progressively develops, this more flexible mechanism can be used to learn non-adjacent linguistic dependencies. This allows filtering out irrelevant information and selectively focusing on relevant elements that reliably predict forthcoming information. As we will argue and support with evidence from infant development, changes in these two different aspects of cognition are not independent. In other words, the attention mechanisms available early on limit the type of linguistic information that infants can extract from speech. The delay in development of more controlled mechanisms of endogenous attention may not indicate a disadvantage in language acquisition but rather an advantage at early linguistic stages. That is, in agreement with Newport’s “Less is More” hypothesis (Newport, 1990), this delay in the development of endogenous attention and the initial use of more automatic exogenous attention mechanisms may allow young infants to face a perceptual simplification of the complex speech stream early in the learning process. Therefore, relying in prosodic cues may be crucial and beneficial during the first months of life, when exogenous attention is the main mechanism available. Such a pattern would lead to the observed early segmentation and acquisition of words during the first months, which is followed by a shift later in infancy to focus on the upcoming information indicated by relevant cues (as in non-adjacent dependency learning) when the infant is able to select which information to attend and which to ignore.

Crucially, the other important difference from the previous theoretical approaches is that our proposal is based on the allocation of attention in the *temporal domain*. Whereas previous proposals have focused purely on visual attention and how it influences the conceptualization of the message to be conveyed (Levelt, 1989) and how this is reflected in the linguistic output, we are interested in how attention affects the processing of the ongoing auditory stimulation -the speech flow. Given that speech is a sequence of sounds that unfolds in time, attention to speech is necessarily oriented in the temporal domain. Because temporal-selective attention directs resources to certain moments in time, enhancing perception (Correa et al., 2006; Nobre et al., 2007), it can allow for the extraction of different events in speech (e.g., consonants, vowels, words, and phrases) that have different durations and appear in a certain order and moments in ongoing speech. Recently, more general proposals have also underscored that cerebral mechanisms for timing and ordinal knowledge are in charge of the neural representation of sequences in different domains, including language (Dehaene et al., 2015). More precisely, speech has temporally rhythmic and salient prosodic cues that capture attention automatically when they appear, helping, for example, to locate boundaries to segment words. On the other hand, segments carrying cues for rule dependencies may have different durations and different onset times (such as suffixes and pronouns). Attention can be progressively tuned to focus on these cues when they are progressively noticed to predict later upcoming dependencies in a sequence of words. This tuning requires the engagement of endogenous attention in the temporal domain. Therefore, acquiring words and rules may require the engagement of different attentional systems. A dynamic shift between systems should develop in the course of learning. Indeed, recent data in adults show that the same prosodic cues can lead to exogenous effects related to segmentation, even in the absence of any possible learning and endogenous effects when the prosodic information can be used as a cue to extract non-adjacent rules (de Diego-Balaguer et al., 2015).

By studying the attention mechanisms involved in language learning, we also pave the way to understand some of the sources of language learning disabilities. In the following sections, we first describe the typically developmental trajectory of attention and language functions and their underlying brain development. We review evidence supporting the hypothesis that maturation of the attention mechanisms may serve as a scaffold for language development, and we review evidence indicating the close relationship between attention deficits and impairments in language acquisition.

## Stages of Development of the Attention System

Before entering into the details of attention development, some conceptual clarifications can be helpful concerning the terms that are used throughout this paper. In the attention literature, a distinction between exogenous (bottom–up) and endogenous (top–down) attention has been classically proposed, and a plethora of studies have dissected the effects that characterize each of these systems and their interactions (Chica et al., 2013). In brief, both types of attention have been proven to facilitate processing. However, exogenous orienting appears even when a secondary task is performed, and it can be voluntary attenuated but not completely suppressed. Endogenous attention is often voluntary, but it can also appear with no effort and even when participants are not aware of the relationship between the cue and the target. Some models of attention (Corbetta and Shulman, 2002; Corbetta et al., 2008) propose a distinction in terms of *stimulus-driven* vs. *goal-directed* attention, which partially overlap with *exogenous* vs. *endogenous* attention but have important discrepancies that are worth mentioning here. Within the frontal and parietal brain regions involved in attention, stimulus-driven attention involves a more ventral fronto-parietal network (Corbetta et al., 2008), including the inferior parietal cortex, the ventral and inferior prefrontal cortex (PFC), and insula, as well as subcortically, the superior colliculus. Goal-directed attention, in contrast, involves a more dorsal fronto-parietal network, including the middle PFC and the superior parietal lobe (Corbetta et al., 2008), and subcortically, the pulvinar of the thalamus.

An important distinction within this framework that might help to understand the attentional systems in a less dichotomic way is the distinction between saliency when (i) no task or goal is present (i.e., exogenous saliency) compared to when (ii) elements are salient because they share some feature that is relevant for the task or goal of the subject despite not being the target of the task (i.e., task-relevant saliency). In other words, a red circle surrounded by green squares will attract attention in the absence of any task due to their exogenous saliency; however, a green circle will drive our attention if our task is to detect a green square because the circle shares a relevant feature (i.e., green) to our task. This distinction is important not only because neuroimaging data show that when performing a task, sensory-salient and task-relevant stimuli induce the activation of different brain networks but also because in the absence of a task, these different types of stimulation do engage the ventral network (Corbetta and Shulman, 2002; Chica et al., 2013). Because very young infants do not have a goal-directed system available, salient stimuli in the environment may trigger the ventral network and subcortical areas. With incremental learning and the progressive availability of the goal-directed system, the relevant elements in the environment attract the ventral attention system in a more *task-relevant* manner. In terms of what this might mean for language, infants may first be attracted by any change in pitch, pauses or in voice onset time in speech sounds due to their intrinsic saliency, whereas later in development, once prosodic characteristics, representations of the phonemes and words of their native language are learned, only those prosodic variations and speech sounds that correspond to the contrast of their language will attract their attention. To avoid misunderstandings in the course of the paper, we will refer to *saliency* to designate only those stimuli that attract attention due to their sensory characteristics irrespective of their relevance.

Turning back to the development of the attentional system, three main attentional mechanisms have been described in the literature on attention development: alertness, orienting, and endogenous attention (Colombo, 2001). The first two characterize exogenous attention. Rudimentary forms of each of the functions of attention are already present to some degree at birth, but each exhibits progressive maturity during the first years of life.

The arousal system is already present at early stages of development. This attention system is associated with an infant’s level of alertness and readiness to process stimuli from the environment. From birth to 2 months of age, alertness is commonly initiated by exogenous stimulation (Wolff, 1965). Very young infants show “obligatory attention” (Stechler and Latz, 1966) or “sticky fixation” (Hood, 1995), a difficulty in interrupting gaze from a given stimulus they are fixating in order to shift attention to a different one. The efficiency of disengaging from and shifting gaze to a stimulus increases during the first months after birth (Hunnius and Geuze, 2004). This phenomenon is tied to the neurological maturation of the visual pathway and associated with subcortical structures (Richards et al., 2010) that, although present at birth, are still developing in terms of their connectivity to most cortical areas (Casey et al., 2004; Uylings, 2006). Between 2 and 3 months of age, maturation moderates the inhibition mechanisms that limit eye-movements, which start to gain cortical control. Effective visual exploration requires disengaging and shifting gaze across different locations, and the perseveration of this sticky phenomenon at 7 months of age is an early feature of later emerging autism (Elison et al., 2013) that persists with disengagement difficulties in childhood (Landry and Bryson, 2004). Therefore, this attentional mechanism might be more related to communicative and social aspects of language development.

At later stages, infants start developing the ability to orient attention toward a particular stimulus in space (Courage and Richards, 2008). The infant’s visual behavior in the first year of life is dominated by an orienting system of attention with two main components (Ruff and Rothbart, 1996). On the one hand, the spatial-orienting network, which includes the posterior parietal cortex and several subcortical systems, mediates attentional functions, such as engagement, disengagement, shifting, and inhibition of return. On the other hand, the object recognition network, which includes pathways from the primary visual cortex to the parietal cortex and the inferior temporal cortex, orients attention to object features. A remarkable developmental progression of this orienting system occurs between 3 and 9 months of age (Ruff and Rothbart, 1996). During this period infant flexibly and quickly orient attention to stimuli in the environment in terms of experiential factors (e.g., novelty or complexity) rather than their exogenous salience (Courage et al., 2006).

Finally, the endogenous orienting of attention shows a slower and later developmental time course than other attention systems, showing a remarkable change during the later parts of the first year and beyond (Colombo, 2001). It is not until the end of the first year that more complex features arise and endogenous control of attention starts acquiring an executive component to a greater extent (Courage and Richards, 2008), which is closely related to the initial maturation of the dorsal prefrontal and the anterior cingulate cortices (Posner and Petersen, 1990). Age-related attentional improvements are related to changes in structural and functional connectivity (Rueda et al., 2015). Data reveal that increased attentional performance is related to greater information transfer in the brain, which involves distributed brain nodes and paths that connect these nodes. Crucially, the anterior cingulate cortex does not begin to develop long-range connectivity with other brain areas until after the first year of life, developing progressively during childhood (Fair et al., 2009; Gao et al., 2009). However, although the structural connectivity pattern in children resembles that of adults, the functional connectivity of attentional networks shows different patterns. While adults’ orienting and executive attentional systems exhibit separate functional networks, these systems are more unified in children (Fair et al., 2007). In summary, whereas exogenous attention shows an earlier maturation course, endogenous attention develops later and slower, continuing its development through childhood and until adolescence (Colombo and Cheatham, 2006; Johnson et al., 2015).

**FIGURE 1:**
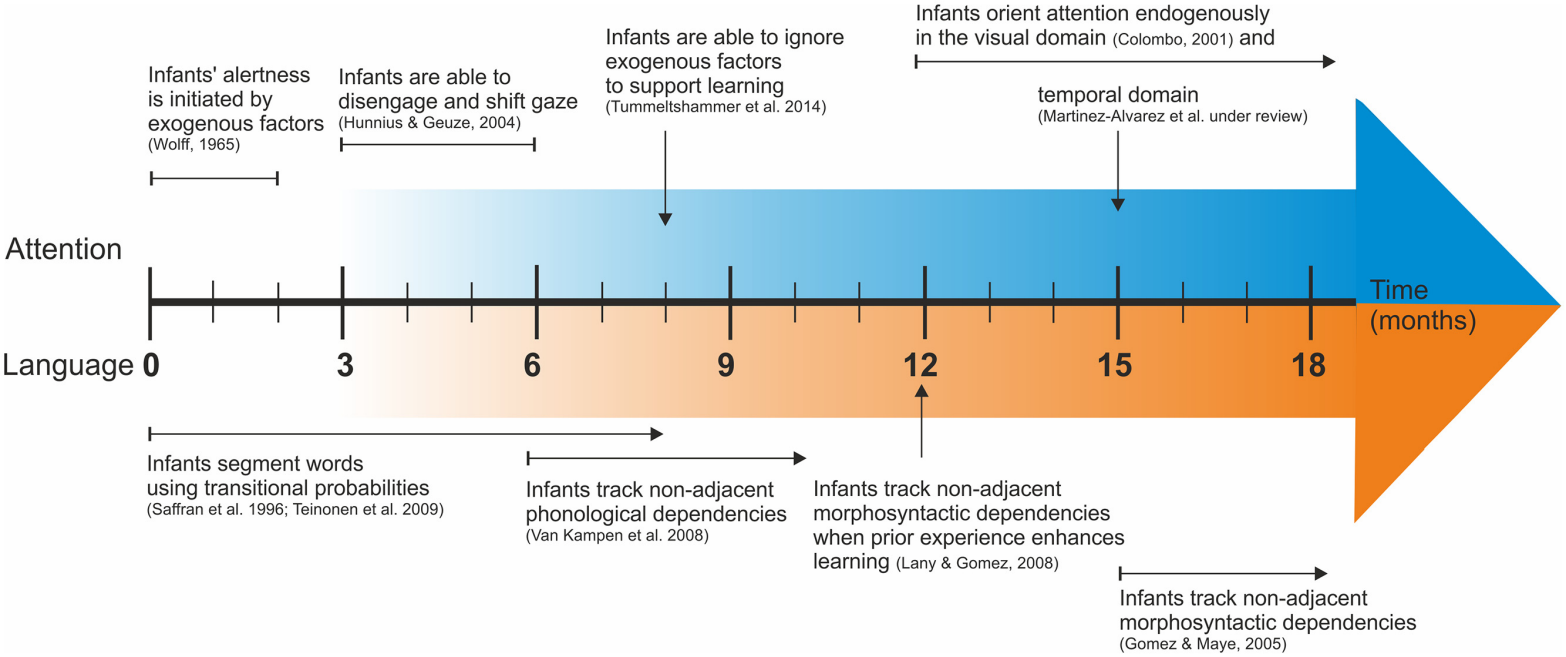
**Timeline of the main maturational milestones in the attention and language domains**. The color gradient in the arrows indicates the progressive development of each domain.

Despite the apparent dichotomy between exogenous and endogenous attention, the appearance of the latter does not imply an inhibition of the exogenous mechanism but a better interaction between both. As it has been highlighted in the adult literature (Corbetta et al., 2008), stimulus-driven attention is able to break the engagement of goal-directed attention, highlighting the close interaction between systems. However, goal-directed attention can attenuate the interference from distractors by decreasing the activation of stimulus-driven attention. The ability to ignore salient distractors to support learning is observed at 8 and 12 months (Althaus and Mareschal, 2012; Tummeltshammer et al., 2014) but is not present earlier, indicating a stronger influence of exogenous factors on young infants’ attention. From this point of view, it is clear that the development of the exogenous and endogenous mechanisms of attention do not show a strict sequential order but rather a smooth overlap with subtle signs of endogenous attention appearing before 8 months of age but with poor command observed to progressive control reached at the end of the first year (see **Figure 1**).

Most of these developmental descriptions of infants have been based on studies of visuospatial attention. Although this description is useful in understanding the developmental progression of attention in general, tracking auditory information in time is critical when we consider language learning due to the intrinsic temporal characteristics of speech. Generally speaking, to orient attention in time, different capacities need to be in place. Infants need to be able to perceive the difference between distinct temporal lags and be sensitive to the order of elements in a sequence. Once these perceptual capacities are available, the attentional system should be able to orient to these different elements in the auditory domain to extract information from speech. Temporal processing is supported by a cortico-subcortical network, including the premotor cortex, basal ganglia and cerebellum that have been proposed to also be involved in speech processing (Kotz and Schwartze, 2010). As previously mentioned, subcortical structures are functional and are used by infants from birth (Casey et al., 2004; Uylings, 2006), and sensorimotor cortices are the first to develop (Dehaene-Lambertz and Spelke, 2015). The early availability of these structures may allow infants to use temporal information in an exogenous manner in the early stages of development.

Studies exploring infants’ ability to perceive time mostly focus on regular temporal structure perception (e.g., rhythm and regular isochronic sequences), i.e., focusing in attention mechanisms that are mainly exogenous and stimulus-driven (Demany et al., 1977; Haith et al., 1988; Adler et al., 2008). These studies show that in the first months of age, infants are sensitive and can orient their attention in time following regular patterns. vanMarle and Wynn (2006) reported that 6-month-old infants can discriminate event durations between 2 and 4 s. Brannon et al. (2004, 2008) additionally showed that 10-month-old infants can detect changes in temporal rhythm by detecting a temporal deviation in a stream of tones formed by a regular inter-stimulus interval. In terms of infants’ ability to benefit from rhythmic and regular patterns, their behavior is similar to that observed in adult research (Large and Jones, 1999; Barnes and Jones, 2000; Sanabria et al., 2011).

In contrast, the ability to orient attention in time endogenously has not been reported in infancy. Recent data (Martinez-Alvarez et al., under review) indicate that the ability to endogenously orient attention in time appears after the ability to orient attention in space. More precisely, whereas 12-month-olds show only spatial orienting abilities, 15-month-olds are able to adapt their anticipatory behavior according to both spatial and temporal predictive cues. A recent study with children revealed also that the developmental trajectory of voluntarily use temporal cues is delayed relative to the use of spatial cues. However, this study showed that 11-year-olds were only able to implicitly but not voluntarily orient attention in time (Johnson et al., 2015), which apparently seems to contradict the results with infants (Martinez-Alvarez et al., under review). Different explanations of these results with children are possible. As Johnson and colleagues explain, one possibility is that the temporal cues were conceptually more demanding than the spatial cues. Another possibility is that the spatial uncertainty of target appearance in their paradigm diminished the utility of the temporal cue. Electrophysiological and behavioral investigations have shown that temporal predictability is most successful when joined with spatial predictability (Doherty et al., 2005; Rohenkohl et al., 2014). Indeed, preliminary evidence from Coull’s lab shows that children can use temporal cues when the spatial location of the target is known in advance (Johnson et al., 2015). Further investigations are needed for a better understanding the development of temporal attention at the functional and anatomical levels.

In sum, the development of attention is characterized by a shift from exogenous, stimulus-driven orienting of attention, particularly during the first 3 months of age, to a smooth progression to greater endogenous control, the first hints observable before 8 months and showing a marked dominance after the first year (Johnson, 1990; Ruff and Rothbart, 1996). Although little evidence is available from attention orienting in the temporal domain, clear effects of sensitivity to temporal differences and rhythmic cues are present early on, whereas endogenous orienting of attention in time appears later.

## Language Development in Infancy in Relation to Attention

In the current section, we review the developmental trajectory of linguistic abilities in infants, focusing on studies on word segmentation and non-adjacent rule learning, which are the two main milestones of interest for our hypothesis. Throughout the review, we point out the role of the attention mechanisms related to the language data available at each stage.

### Early Stages of Language Learning and Exogenous Mechanisms of Attention

The early capacities of infants to acquire their native language have been extensively reported. Even before they begin to produce their first words, infants have already acquired an important amount of linguistic knowledge. One very early ability is their sensitivity to perceive the rhythmic characteristics of language at birth, showing discrimination of the stress patterns in different languages and an early preference for their native language stress pattern (Nazzi et al., 1998). Importantly, prosodic characteristics, such as intonation, stress and pitch variations, are salient perceptual cues that can easily attract infant’s exogenous attention. These prosodic cues play a key role in word segmentation because infants exploit these even before they use other cues to locate word boundaries (Mattys et al., 1999).

During the first months of life, infants are capable of extracting words in spoken language by detecting and exploiting other perceptual cues. For example, neonates (Teinonen et al., 2009) and 8 months old infants can make use of statistical regularities between adjacent syllables (also known as transitional probabilities, TP) to locate word boundaries and extract words from both artificial (Saffran et al., 1996) and natural languages (Pelucchi et al., 2009). On the other hand, the combination of both prosodic and statistical cues shows how predominant are the acoustic features of infant-directed (ID) speech (e.g., exaggerated pitch contours) compared to adult-directed (AD) speech to attract infants’ attention (Fernald, 1985; Cooper and Aslin, 1990) and to facilitate infants’ word segmentation (Trainor and Desjardins, 2002; Thiessen et al., 2005).

Statistical learning is a remarkable ability, and numerous studies have been developed to understand the mechanisms underlying and the factors affecting this type of learning. Indeed, several important features of growing literature with the same paradigms are important to mention in relation to the hypothesis outlined here. One critical factor is that statistical learning is a simple adaptive capacity that can also be found in other animals that have much less developed prefrontal cortices. For example, rodents exposed to the same type of linguistic speech streams are able to correctly segment it, albeit with a somewhat different computation (Toro and Trobalón, 2005). Another important feature is that the presence of statistical regularities in the input captures attention (Turk-Browne et al., 2005). Thus, even when no effort to learn is given, regularities can be extracted from the input (Saffran et al., 1997), capturing our attention in an automatic manner, which is consistent with the fact that even newborns are able to detect these statistical regularities (Teinonen et al., 2009). Therefore, the development of endogenous attention is not necessary for this learning to occur. In the same vein, electrophysiological evidence indicates that once words are segmented, the recognition/detection of a known word within the speech stream also captures attention (Sanders et al., 2002; Parise et al., 2010; de Diego-Balaguer et al., 2015), enhancing the long-term memorization of the segmented word forms.

Another important fact underscores the importance of exogenous attention in these early learning stages and highlights the adaptive function of the unavailability of the endogenous system in young infants. In adults, the manipulation of diverted attention, orienting endogenous attention outside the speech stream, can interfere with teach (Toro et al., 2005). Adults and older infants can orient their attention endogenously, and although this can be helpful when it converges to track the critical information for learning, it can also interfere with learning when it diverts from the correct focus of attention. For example, attention diverted from the dependency by the attraction of novel words that need to be ignored prevents non-adjacent learning. Infants’ ability to generalize the detection of non-adjacent dependency to nonsense stems (e.g., ***These*** *meep****s***) occurs only if they are first presented with familiar stems (e.g., ***These*** *chair****s***). When attention is captured by a *novel* intervening element, learning of non-adjacent dependencies is altered (Soderstrom et al., 2002). In contrast, if salient information automatically captures infants’ attention and this information is helpful for learning, the absence of endogenous attention prevents infants from disengaging and reorienting their attention to a different focus of attention that may interfere with the correct computation. In this way, the early dominance of this automatic exogenous mechanism can make learning more likely to occur. Other salient features, such as adjacent repetitions, can also act as important attentional attractors improving learning. Already present at birth, infants possess an automatic perceptual mechanism to detect repetitions in the auditory domain (Endress et al., 2009). This is reflected in greater activation in the temporal and left frontal brain areas when tested for recognition after exposure to simple repetition-based structures (ABB; e.g., “mubaba,” “penana”) than to random sequences (ABC; e.g., “mubage,” “penaku”).

Overall, the evidence indicates that the characteristics of speech with their statistical regularities and the salient prosodic cues are perfectly adapted to make the most of the early availability of exogenous attention. By engaging exogenous stimulus-driven attention, available since birth, learning can be achieved. The absence of control of voluntary attention at these early stages of development does not limit infants’ ability to acquire language but rather helps them by allowing infants to follow their stimulus-driven mechanism to capture the relevant information for learning automatically.

### Early Signs of Non-Adjacent Dependency Learning in the Rise of Endogenous Attention

Although the ability to segment and extract words from speech is a critical milestone of language acquisition, to acquire the grammar of their language infants must also track non-adjacent relationships. Importantly, the extraction of hierarchical structures relies on temporally distant relationships and is fundamental to capture the properties of language (Chomsky, 1957). Nonadjacent dependencies refer to cases in which two elements co-occur over one or more intervening elements. In natural languages, for example, in English, there is an association between auxiliaries and inflectional morphemes, irrespective of the intervening verb stem (e.g., **is** walk**ing**; **is** runn**ing**; **is** eat**ing**). Infants must dismiss the variable irrelevant information and focus instead on the invariant relevant cues that predict the non-adjacent dependency (Gómez and Maye, 2005).

Because the endogenous system appears progressively in the course of development, its initial use in its earliest stages depends on the convergent presence of exogenous cues. The first signs of non-adjacent tracking in language are observed in the phonological domain where the presence of exogenous cues helps infants to track the dependencies grouped by their high similarity (for a review, see Sandoval and Gómez, 2013). For example, infants as young as 7 months can use harmony on vowel which are more salient than consonants and linked to prosodic variations as a cue to find word boundaries (Mintz and Walker, 2006; Kanpem et al., 2008) but cannot use consonantal harmony (Nazzi et al., 2009; Gonzalez-Gomez and Nazzi, 2012). They need to reach 10 months of age before they can, an age where endogenous attention starts to be more prominent. In a similar vein, newborns can discriminate adjacent rules based on the repetition of the same syllable but not when rules are non-adjacent (ABA; e.g., “bamuba,” “napena”) (Gervain et al., 2008). In contrast, 7-month-old and older infants track non-adjacent dependencies but only under some circumstances; when non-adjacent syllables are identical and the interleaved syllables are different (e.g., *le di le, ga po ga)* (Marcus et al., 1999; Gerken, 2006). Unexpectedly, a more recent study demonstrated that German infants as young as 4 months of age could discriminate between grammatical and ungrammatical non-adjacent dependencies in Italian (Friederici et al., 2011). As the authors indicate, Italian morphosyntactic dependencies also contain phonological dependencies. Given that phonological, non-adjacent dependencies are tracked from very early stages in development, it has been proposed that 4-month-olds may be tracking the phonological aspects to discriminate these non-adjacent dependencies. In other words, the exogenous attentional resources already available to 4-month-olds could have driven the success of such young infants in this task.

### Learning more Challenging Non-Adjacent Dependencies with Greater Maturation of Endogenous Attention

Although young infants can track non-adjacent linguistic rules under certain learning conditions (e.g., when the dependent units are similar and the intermediate elements are dissimilar), learning of morphosyntactic dependency appears several months after phonological dependency learning has occurred. This non-adjacent dependency learning that appears in more challenging perceptual and linguistic arrangements, requires greater involvement of endogenous mechanisms. Simply tracking non-adjacent dependencies can be used to locate word boundaries but is not enough to extract and generalize the underlying rule that entails the creation of abstract categories for generalization (Peña et al., 2002).

In this context, prosodic information in natural languages provides reliable cues not only for word segmentation but also for rule learning (Jusczyk, 2002) because prosodic pauses tend to co-occur with syntactic boundaries. Nevertheless, although prosodic cues play a role in word segmentation from birth, it is not until the first year that infants start exploiting these cues for rule extraction (Johnson, 2008; Seidl and Johnson, 2008). The presence of this prosodic information in an artificial language enhances the extraction of non-adjacent dependencies compared to continuous speech streams without pauses (Peña et al., 2002). An important point to highlight is that the presence of pauses *per se* does not improve learning of non-adjacent dependencies. Those pauses need to occur at the boundaries of the position of the dependencies to be useful (Endress et al., 2005; Mueller et al., 2010). The use of these cues (stress pattern or prosodic pauses) for word segmentation requires only orienting attention to the position of the prosodic information that captured attention, that is, in an exogenous fashion. However, the use of prosodic pauses for rule extraction additionally requires the use of this cue to selectively focus attention on concurrent phonological information at this specific position. This cue then has to be used as a relevant predictor of forthcoming information to extract the rule dependency, which implies focusing attention to this cue and the predicted element while disregarding the intervening irrelevant information.

Within the morphosyntactic domain, nonadjacent relationships are often found between subject and verb agreement (**he** walk**s**) or between auxiliary and verb agreement (he **is** walk**ing**). Learning of morphosyntactic, non-adjacent dependencies emerges after the first year of life (Gómez and Maye, 2005). This developmental course is reasonable when considering the challenge of the task, that is, in order to track the dependency among non-adjacent elements, infants must first identify the morphemes without involving any given similarity and then track the dependency between them across intervening elements irrelevant to the rule dependency (**he** walk**s**; **he** run**s**; **he** eat**s**). In one of the first studies exploring infants’ ability to learn verb–tense agreement (Santelmann and Jusczyk, 1998), researchers reported that 18-month-olds accepted grammatical phrases in English, such as “is running,” and rejected ungrammatical phrases, such as “can running,” whereas 15-month-olds were not able to differentiate between the phrases. Moreover, learning was possible only under certain conditions, with infants succeeding when the intervening element extended three syllables or less (e.g., *Grandma* ***is*** *always sing****ing***, but not *Grandma* ***is*** *almost always sing****ing***).

In addition, in order to learn a non-adjacent relation of the form “**these** cat**s,**” infants must track a dependency between two elements that occur over an intervening element and create different categories (e.g., determiner, noun, verb). Several lines of research have explored the mechanisms underlying the ability of grouping elements into categories. For example, it has been proposed that *frequent frames* (e.g., “these × are”) yield category formation by their frequent co-occurrence with intervening content words and constitute the basis for the creation of grammatical categories (Mintz, 2003). Gómez and Maye (2005) showed that 15- and 18-month-old succeed when frames have high variability in the intervening word inside the frame but failed with low variability which is in agreement with the frame-based categorization proposed by Mintz (2003). Similarly, increasing the variability of the irrelevant intervening information makes adjacent relations less statistically informative and the non-adjacent dependency more prominent allowing learners to focus on the relevant and reliable relationship among non-adjacent elements (Gómez, 2002). Interestingly these different studies converge in a similar age between 15 and 18 months old as Gerken et al. (2011) where these authors found that infants use selective attention to focus on languages having learnable grammatical patterns. These studies converge to the parallelism between the development of non-adjacent dependency learning, category formation and endogenous control of attention in the second year of life. The importance of correct tuning in attention for the acquisition of non-adjacent rules is also seen in the Lany and Gómez (2008) study, where infants younger than those of the previous studies were able to track non-adjacent dependencies if the correct attention focus was guided by training them first on the dependencies between categories. Infants later discriminated grammatical and ungrammatical items involving non-adjacent dependencies with the same category words.

Thus, the overall pattern in agreement with the progressive ability to orient attention endogenously and the close collaboration between exogenous and endogenous attention. Early on, infants need more concurrent exogenous cues such as high degree of similarity, same identity between the dependent pairs (Creel et al., 2004; Onnis et al., 2005) or prior exposure to them (Lany and Gómez, 2008; Lai and Poletiek, 2011) (for a review, see Perruchet et al., 2012), to help them to orient their attention to the relevant information (Pacton and Perruchet, 2008; Pacton et al., 2015), allowing a greater interaction between exogenous and endogenous attention. At later stages of development, after the first year of age, the improved endogenous system allows infants to rely less on the availability of these salient features to orient their attention to the relevant information.

An important point to consider is that signs of discrimination of more complex non-adjacent dependencies at a very early age have been observed only in electrophysiological studies (Mueller et al., 2012). Online EEG measures may not reflect the same knowledge as more overt behavioral responses that require greater explicit knowledge. In that sense, it is worth considering that indicators of prediction present from birth are reflected in mismatched responses in the EEG at the presentation of unexpected events, and these early online effects reflect these more automatic prediction mechanisms. However, recent research has shown that electrophysiological indexes of conscious access, equivalent to the P300 in adults, that show a non-linear pattern, can only be tracked clearly at the end of the first year. This response associated to consciousness was visible and sustained from 12 to 15 months of age (750 ms) and may serve to amplify the sensory input through selective attention (Kouider et al., 2013). Conscious access before the first year of age may not be possible because even if the structural architecture is in place, its immaturity may not allow an adequate flux of information for conscious availability (Dehaene-Lambertz and Spelke, 2015). From the perspective presented here, this conscious access may be required for these predictions to reach a long-lasting representation that may allow the infant to show behavioral effects. More studies are needed to examine early computation of different types of non-adjacent dependencies in infancy. New research should take into account the role that variables attracting attention may have in their acquisition in order to understand when the capacity actually arises in development.

## Brain Development of the Attention and Language Networks

**FIGURE 2:**
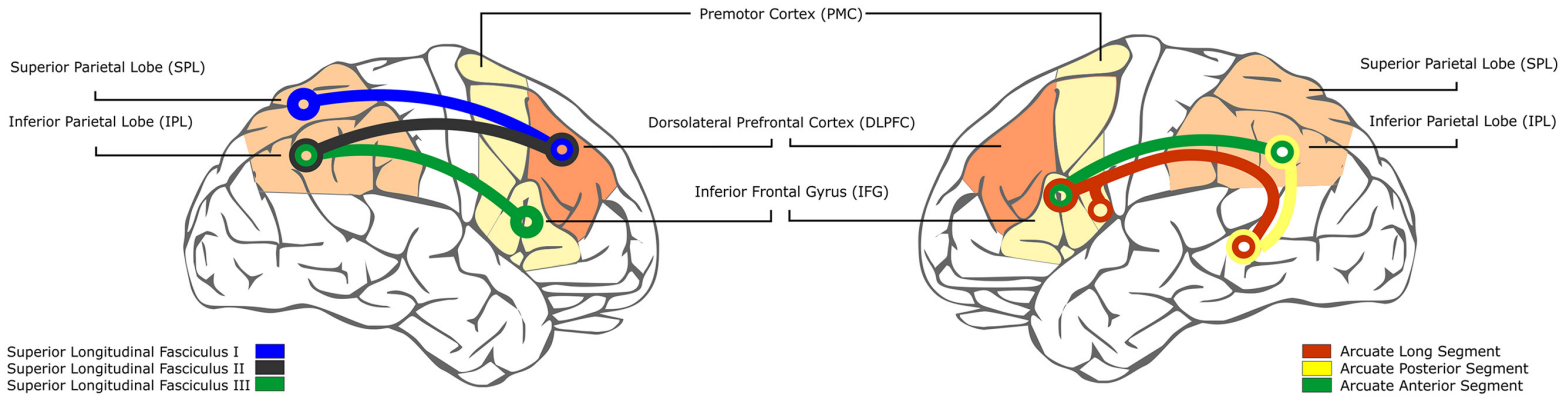
**Schematic representation of the fiber tracks forming the dorsal and ventral attention networks and the dorsal language pathway**. **(Left)**: Representation of the three branches of the superior longitudinal fasciculus in the right hemisphere. **(Right)**: Representation of the three segments of the arcuate fasciculus in the left hemisphere. The different color codes in the lobules of interest are shown in a gradient from the earliest to mature (lighter color) to the latter to mature (darker color).

In terms of brain development, the parallel maturation of the attention and language network is also evident. This is in part unavoidable given the partial overlap between those two networks. As we have previously mentioned, a fronto-parietal network with either more ventral or dorsal distribution is related to stimulus-driven and goal-directed attention mechanisms, respectively. These areas are connected through the superior longitudinal fascicle (SLF), and the ventral and dorsal connectivity is ensured through the SLF III branch and I branch of this fascicle, respectively; these two connections have been proposed to interact through the SLF II, which connects the dorsal regions of the PFC to the ventral regions of the parietal lobe (de Schotten et al., 2011; **Figure 2**, left).

For language, a division in ventral and dorsal pathways ensures audio-motor integration and language production dorsally and language comprehension and semantic processing ventrally (Hickok and Poeppel, 2007). Direct connections between the language-related areas in the left frontal and temporal cortices are sustained dorsally through the arcuate fasciculus (AF) and ventrally by paths running through the extreme capsule (Saur et al., 2008; Brauer et al., 2011, 2013). The dorsal connection is also assured indirectly through the parietal lobe with shorter segments (Catani et al., 2005): an anterior segment connecting the premotor and inferior frontal regions with the IPL and a posterior segment connecting inferior parietal and temporal cortices (**Figure 2**, right). There is some controversy concerning the terminations of the AF (Dick and Tremblay, 2012, for a review). Interestingly, this bundle overlaps with the SLF III, previously mentioned in relation to the ventral attention network, the anterior segment of the AF and the SLF III having a greater right lateralization (Catani et al., 2005; López-Barroso et al., 2013). Although the same nomenclature is used for attention and language in terms of ventral and dorsal streams, only the ventral attention and dorsal language stream overlap (see **Figure 2**). Although we have based this section on models of attention based on visual attention, Corbetta et al. (2008) did mention that the ventral attention network responds to different modalities. The overlap between the ventral attention and dorsal language networks is even greater if we consider that the temporal attention network shows a greater left functional lateralization, pointing again to the importance of temporal attention in speech processing (Coull et al., 2011).

During development, studies of whole ventral language connections demonstrated that newborns exhibit an adult-like ventral connection between the frontal and temporal lobes, and even children at 7 years of age have a preferential use of this pathway for sentence comprehension, in contrast to adults, who preferentially use the dorsal pathway (Brauer et al., 2011). In contrast, the dorsal pathway follows different developmental courses, with two subparts maturing at different rates. Whereas the dorsal connections reaching the premotor cortex are functional at birth, the terminations of the dorsal pathway reaching the posterior portion of Broca’s area (BA 44) are still underdeveloped (Perani et al., 2011) and are not fully myelinated at the age of seven (Brauer et al., 2011, 2013). Adult studies on the learning of new languages indicate that whereas the audio-motor subpart is related to word learning (López-Barroso et al., 2013), the processing of non-adjacent elements relies on the latter subpart running from frontal BA 44 (Friederici, 2011). This pathway, which may support hierarchical (non-local) dependencies (Boeckx et al., 2014), follows a later and slower rate of development, similarly to non-adjacent, dependency rule learning.

Rapid changes are observed during the first year of life in terms of maturation. Sensory and motor systems myelinate earlier than brain systems serving higher level functions (Flechsig, 1920). Myelination starts at different times and occurs at different rates in different areas. At birth, there is little differentiation between gray and white matter in cortical areas. The primary visual cortex rapidly matures during the first 3 months with parallel myelination of optical radiations, whereas the primary auditory cortex and acoustic radiations extend over the first 3 years of life. The frontal areas and cortico-cortical connections continue to mature until puberty, but myelination is already observed during the first year in all associative regions. Diffusion measures increase with the compactness/myelination of the tracts in the left lower part of the cortico-spinal tract and in the parietal part of the AF relative to the right during the first 3 months of life. During this period the maturation of the right hemisphere is generally faster than the left (i.e., superior temporal sulcus, STS), but the inferior frontal gyrus (IFG) shows earlier left than right development. The left AF matures faster than the right and correlates with the maturation of BA 44 and the posterior part of the STS (Dehaene-Lambertz and Spelke, 2015 for a review).

Postnatal maturation shows subcortical white matter expansion in the connections to the frontal, anterior temporal, and parietal cortices, as measured by diffusion imaging and volume expansion (Hill et al., 2010). Surface expansion reflects an underlying change in synaptogenesis, dendritic arborization, gliogenesis, and intracortical myelination. The lateral temporal and parietal lobes and the dorsal and medial prefrontal regions are functionally and structurally not mature at birth. They show high expansion in cortical folding in both hemispheres in infants compared with adults. The latest maturation in synaptic density, peak cortical thickness, and mature values of gray matter density are reached in the dorsolateral prefrontal cortex. The comparison between human and macaque monkey cortices reveals that these dorsal, medial frontal and lateral parietal cortices show correlated high postnatal and evolutionary expansion (Hill et al., 2010). This pattern suggests similar patterns of cortical expansion in the development and evolution of these areas, which points to the importance of these areas for human specific functions.

These changes in connectivity at the structural level are also reflected in functional connectivity. Graph-theoretic measures of infants’ brains (Power et al., 2010) indicate that the developing functional networks are in some respects similar to adult networks. The necessary connections are present; however, the brain connectivity compared to adults tends to have strong resting state functional connectivity MRI (rs-fcMRI) signal correlations with nearby regions, even during childhood. The progressively local correlations tend to weaken, whereas correlations with more distant regions, such as those between the frontal and parietal cortices, tend to increase. This trend stems from synaptic pruning that contributes to reduced local rs-fcMRI correlation, and myelination that could facilitate increased long-range connectivity.

Considering the overall attention and language networks, brain regions and connections of overlap are observed between the ventral attention network and the dorsal language network (**Figure 2**). Ventral prefrontal and insular regions integrating the ventral attention network and the anterior segment of the AF show an early availability, whereas the IPL and their connections show a later and more progressive development. This delayed development also affects the dorsal attention network with the dorsal prefrontal regions having a slow maturation extending to childhood and with delayed maturation of the parietal lobe (Casey et al., 2000; Fuster, 2001). There is evidence showing that the left IFG is engaged in the extraction of TPs (Karuza et al., 2013) as well as the PMC (Cunillera et al., 2009) when no other cue is available to segment speech (McNealy et al., 2006; Scott-Van Zeeland et al., 2010). The early functionality of the left IFG and the premotor cortex (PMC) allows early use of TPs and stimulus-driven attention to orient to salient prosodic information and to segment speech. When the dorsal prefrontal cortex starts to be maturationally functional during the second year of life (Colombo and Cheatham, 2006), the dorsal fronto-parietal network allows for more proficient control of attention. The later maturation of the dorsal prefrontal cortex (DLPFC) and part of the ventral attention network (i.e., IPL), including the temporo-parietal junction (TPJ) (see **Figure 2**), allows progressively to (i) orient the ventral attention network to task-relevant representations (e.g., phonemes of the native language and segmented words) created in the earlier stages of development, (ii) recruit goal-directed attention, (iii) optimal functioning of the attention system, that requires the effective interaction between the two networks through the TPJ and the DLPFC (Corbetta and Shulman, 2002; Corbetta et al., 2008), necessary to accurately and selectively attend to specific stimuli and shift the focus of attention when relevant stimulation appears.

## Attention Deficits and Language Development Disorders

The proposal delineated here makes a straightforward prediction in relation to the effects of attention deficits in language development. If control of attention is a function used for the optimal acquisition of non-adjacent rule dependencies, then impairments in the development of this function should interfere with the acquisition of these rules. In contrast, early language development relying on more automatic attention mechanisms should not be affected.

Commonly, children acquire language rapidly and effortlessly. However, some children show problems acquiring language. Specific language impairment (SLI) is classically defined as a developmental disorder of language characterized by difficulty in acquiring language in the absence of neurological damage, hearing deficits, or intellectual disabilities (Bishop, 1992; Leonard, 1998). The prevalence of SLI in pre-school children is approximately 7% (Tomblin et al., 1997; Law et al., 2000). Longitudinal studies reveal that more than 70% of diagnosed cases of SLI in kindergarten persist into adulthood (Johnson et al., 1999). SLI children have been shown to have difficulties in the acquisition of non-adjacent dependencies (Hsu et al., 2014) and in the use of prosodic information for syntactic processing (Sabisch et al., 2009). In a longitudinal study, impaired prosodic processing of word stress during early development was shown to be an early marker of risk for SLI (Weber et al., 2005).

Linguistic impairments often co-occur with non-linguistic deficits, including attention-deficit/hyperactivity disorder (ADHD). Both SLI and ADHD frequently overlap within the same children, that is, comorbidity between the two disorders is commonly found (Baker and Cantwell, 1992; Benasich et al., 1993; Coster et al., 1999; Noterdaeme and Amorosa, 1999; Tomblin et al., 2000; Lindsay et al., 2007). ADHD is the most frequent diagnosis among children with language impairments (Cohen et al., 2000). Longitudinal studies suggest that SLI children have a profound risk for ADHD (Baker and Cantwell, 1987; Cantwell and Baker, 1987; Beitchman et al., 1989; Benasich et al., 1993; Redmond and Rice, 1998, 2002). More precisely, deficits in selective attention (Stevens et al., 2008) and sustained attention (Spaulding et al., 2008; Finneran et al., 2009) have been found in children with SLI. ADHD is a common childhood disorder characterized by a persistent pattern of inattention and/or developmentally inappropriate levels of hyperactivity/impulsivity (American Psychiatric Association, 2000). ADHD prevalence is approximately 10% in children (Faraone et al., 2003; Pastor and Reuben, 2008). As with SLI, children with ADHD are a highly heterogeneous group. ADHD is commonly divided into three subtypes: ADHD-Inattentive (ADHD-I), ADHD-Hyperactive-Impulsive (ADHD-H/I), and ADHD-Combined type (ADHD-C). Whereas children in the ADHD-I subgroup usually show difficulties with attention control, sustained attention and are often inattentive, ADHD-H children exhibit high levels of activity and poor impulse control. ADHD-I children do poorly in tasks requiring sustained attention, covert shifting of attention and selective attention. Thus, individual differences in the control of selective attention in infancy may be related to ADHD-I outcomes. Children in the ADHD-I group are more probable to meet criteria for learning disability than ADHD-H children (Willcutt and Pennington, 2000).

Similar to the findings on attention deficits found in SLI children, a similar pattern is present in ADHD children. Between 50 and 90% of children with ADHD have co-occurring language difficulties (Gualtieri et al., 1983; Camarata et al., 1988; Love and Thompson, 1988; Tirosh and Cohen, 1998). However, the overlap between these disorders shows an asymmetrical pattern, that is, more ADHD children have co-occurring SLI than SLI children have co-occurring ADHD (Tannock and Schachar, 1996). Higher order cognitive functions (e.g., executive functions, working memory, and attention) have been explored as possible causal deficits for SLI and ADHD disorders (e.g., Cardy et al., 2010; Hutchinson et al., 2012).

In SLI, abnormal diffusion measures are observed systematically in the SLF and AF (Verhoeven et al., 2012; Roberts et al., 2014). A more recent study showed also differences in the ventral language network (i.e., the inferior fronto-occipital fasciculus, IFOF) (Vydrova et al., 2015). The discrepancies between studies may stem from the heterogeneity of the disease with children with more semantico-pragmatic profiles that are more likely to show differences in the IFOF function and those with and without associated ADHD, which may cause an associated SLF abnormality in addition to the AF. The brain structures supporting cognitive functions commonly associated with ADHD have also been investigated. Gross anatomical changes in brain dimensions are often associated with ADHD, specifically, reduced dimensions of the caudate nucleus, the prefrontal cortex, the corpus callosum, and the cerebellar vermis (see Bush et al., 2005 for a review) and in the parietal lobes (Sowell et al., 2003) are found in ADHD. Evidence from pathophysiology research has shown that ADHD physiology involves dopaminergic and noradrenergic pathway dysfunction in the prefrontal cortex and subcortical regions of the brain (Barkley et al., 1992; Castellanos et al., 1996; Faraone and Biederman, 1998; Konrad et al., 2006). This network partially overlaps with both goal-directed attention and temporal processing. DA dysfunction affects mainly the dorsal regions of the PFC, which are those required for goal-directed attention. The subcortical regions affected (i.e., striatum) and the cerebellum are important structures for temporal processing (Coull et al., 2011).

Recent studies have provided the first evidence that temporal selective attention during speech perception predicts language outcome in preschool children. Children who selectively allocate attention to informative moments during speech, such as word onsets, demonstrate better metalinguistic capacity (Astheimer et al., 2014).

## Conclusion

Infants acquire language exceptionally fast and without any given instruction. But, how can infants so easily achieve such a remarkable landmark, whereas adults struggle to do so? Following Kuhl’s view (Kuhl, 2004), understanding how the early brain is committed to the statistical and prosodic patterns experienced early in life helps to explain the long-standing puzzle of why infants are better language learners than adults. One of the possible answers is the way their cognitive development is structured, with functions, such as attention, appearing in an incremental fashion and assisting language learning.

Based on the characteristics of the developmental trajectory of the attention and language systems, we have outlined the hypothesis that attention development, characterized by an initial phase when attention is stimulus-driven, followed by a progressive ability to endogenously control the focus of attention, shapes the developmental trajectory of language. In the evidence reviewed here, we have seen that the learning trajectory of two types of linguistic learning (words and rules) shows a different profile in infant language development. Whereas words in fluent speech are already segmented and extracted at early stages, non-adjacent dependencies occurring over temporally distant elements are learned many months later (see **Figure 1**).

More precisely, the early segmentation and word learning abilities is profoundly influenced by the salient characteristics of the speech signal, with an important role of prosodic information. Later acquisition of more complex information associated with the extraction of more distant dependencies is influenced by variables that help infants to focus attention on the relevant elements carrying the dependency and to disregard the information that is not relevant for the acquisition of the dependencies. This trajectory goes hand in hand with the development of the ability to progressively orient attention endogenously. Early in this phase, infants require more concurrent salient cues, such as phonological similarity or identity repetition, to help them to orient their attention to the relevant information. A greater development of goal-directed attention allows infants to learn less salient, non-adjacent dependencies by relying more on endogenous cues. In terms of brain development, whereas the initial stages of development rely on the availability of some areas of the ventral attention network, including the ventral prefrontal regions and the premotor cortex, the latter stages require the maturation of more dorsal prefrontal and parietal regions (see **Figure 2**).

We consider that this development of attention in different stages allows for an earlier simplification of learning. This early learning is driven by the automatic capture of attention, creating the first building blocks that learning can lean on when control of attention allows for the extraction of more complex relations between non-adjacent elements in speech. Data from adults show that they can track both adjacent and non-adjacent information at the same time, and one information can interfere with the other (Romberg and Saffran, 2013). Thus, the inability to reorient attention away from the automatic attractors of attention is valuable in the early stages of acquisition, allowing for incremental learning.

Moreover, the same exogenous system that allows young infants to extract words using salient cues may also help them to extract complex rules. Young infants are able to succeed in non-adjacent learning that otherwise would not be available after the first year of life. In these early stages, this success of non-adjacent dependency tracking occurs only under certain conditions. Applying our present proposal to this developmental scenario, two main conditions should be fulfilled to extract non-adjacent dependencies in the early stages of development: (1) a rudimentary mechanism of endogenous attention should be available to select certain predictive elements and to disregard irrelevant information, and (2) stimulus-driven factors should be present in the linguistic input (e.g., certain degree of similarity or saliency) to automatically capture the exogenous attention system.

The implications of our hypothesis are clear in terms of the parallelism between the development of the endogenous attention system and the rule learning abilities in healthy infants. This relation is seen not only in healthy development but also in the effects of attention deficits in relation with impairments in language development. The importance of being able to exploit the available information given by exogenous cues, such as prosodic information, to orient attention endogenously is crucial not only in infant healthy development but also in studies with different pathologies.

Comprehending the cognitive processes involved in language development is of critical importance for our understanding of why, under certain conditions, language development impairment occurs. However, research in the field of language development often offers limited explanations bounded within the language domain, ignoring the importance of other cognitive functions. The present proposal overcomes these limits and presents an integrative approach to understand the role of attentional tuning during language acquisition. By reviewing the main stages of attention and language development and possible impairments, we have strengthened the importance of taking an interdisciplinary approach to the study of human development. We believe that this integrative approach exploring the role of temporal attention as a scaffold for language development can lead to a wider scope than previous proposals, allowing the development of a precise model of language and cognitive function interaction during learning that has important clinical and developmental consequences, hence providing an important contribution to the language learning and language rehabilitation fields.

## Author Contributions

RD-B, AM-A provided the ideas and wrote the article. FP contributed to discussions, writing and checked that the infant research review was accurate.

## Conflict of Interest Statement

The authors declare that the research was conducted in the absence of any commercial or financial relationships that could be construed as a potential conflict of interest.
